# Fusion of the Pharyngeal Wall to the Soft Palate Around the Laryngeal Inlet Isolates the Airway in Odontocete Cetaceans

**DOI:** 10.1093/iob/obag012

**Published:** 2026-03-27

**Authors:** A W Vogl, R Cieri, P Palia, R Shadwick, P Cottrell, S Raverty

**Affiliations:** Department of Cellular and Physiological Sciences, Life Sciences Institute, Faculty of Medicine, The University of British Columbia, Vancouver, British Columbia V6T 1Z3, Canada; Department of Zoology, Faculty of Science, The University of British Columbia, Vancouver, British Columbia, Canada; Department of Ecology & Evolutionary Biology, Yale University, New Haven, CT 06520, USA; Department of Cellular and Physiological Sciences, Life Sciences Institute, Faculty of Medicine, The University of British Columbia, Vancouver, British Columbia V6T 1Z3, Canada; Department of Zoology, Faculty of Science, The University of British Columbia, Vancouver, British Columbia, Canada; Department of Fisheries and Oceans Canada, Vancouver, British Columbia, Canada; Animal Health Center, British Columbia Ministry of Agriculture, Abbotsford, British Columbia, Canada

## Abstract

In odontocete cetaceans (toothed whales, dolphins, and porpoises), a palatopharyngeal sphincter permanently anchors the laryngeal inlet in the nasopharynx and isolates the airway from the gut tube. Using an MRI data set of a pacific white-sided dolphin (*Aethalodelphis obliquidens*), together with dissections of the dolphin and two neonatal harbour porpoises (*Phocoena phocoena*), we show that the sphincter is formed by fusion of part of the superior constrictor of the pharyngeal wall to the soft palate. The part of the superior constrictor that participates in forming the sphincter is brought close to the midline by the presence of palatine plates that: (1) project medially from the ventral aspects of the pterygoid bone on each side, (2) lie caudal to the palatine bones of the hard palate where the soft palate normally attaches in other mammals, and (3) divide rostral parts of the soft palate into a part dorsal to the plates and a part ventral to the plates. Based on the presence and the anatomy of major muscles of the soft palate, we present a biomechanical model for how the palatopharyngeal sphincter functions and is positioned during swallowing and breathing. We conclude that the sphincter is most contracted during swallowing to hold the laryngeal inlet in position while large prey items pass through the laryngopharynx, and is most relaxed during breathing when the laryngeal inlet maximumly opens to allow unrestricted airflow.

## Introduction

An important field of study in the evolution and embryology of vertebrates is the development of the lower airway from the gut tube, and in mammals the formation of a secondary bony palate that separates the single buccal cavity into an oral cavity below and nasal cavities above (Parrington and Westoll 1940; Smith 1992). In most mammals, the airway and food pathways cross in the oropharynx and a series of valves ensure that food and liquid do not enter the lower or upper airway during swallowing. Key among these valves is the soft palate that attaches to the caudal end of the hard palate formed by parts of the maxillary and palatine bones. The soft palate can be depressed against the elevated back of the tongue to close off the oral cavity from the oropharynx so that food can be manipulated in the oral cavity while still breathing. During swallowing, the soft palate elevates to close the pharyngeal isthmus between the oral and nasal parts of the pharynx so that material does not enter the upper airway. At the same time, valves (epiglottis and vocal folds) associated with the larynx close the upper end of the lower airway so that food or liquid only enters the esophagus. Most adult mammals cannot breathe and swallow at the same time.

**Fig. 1:**
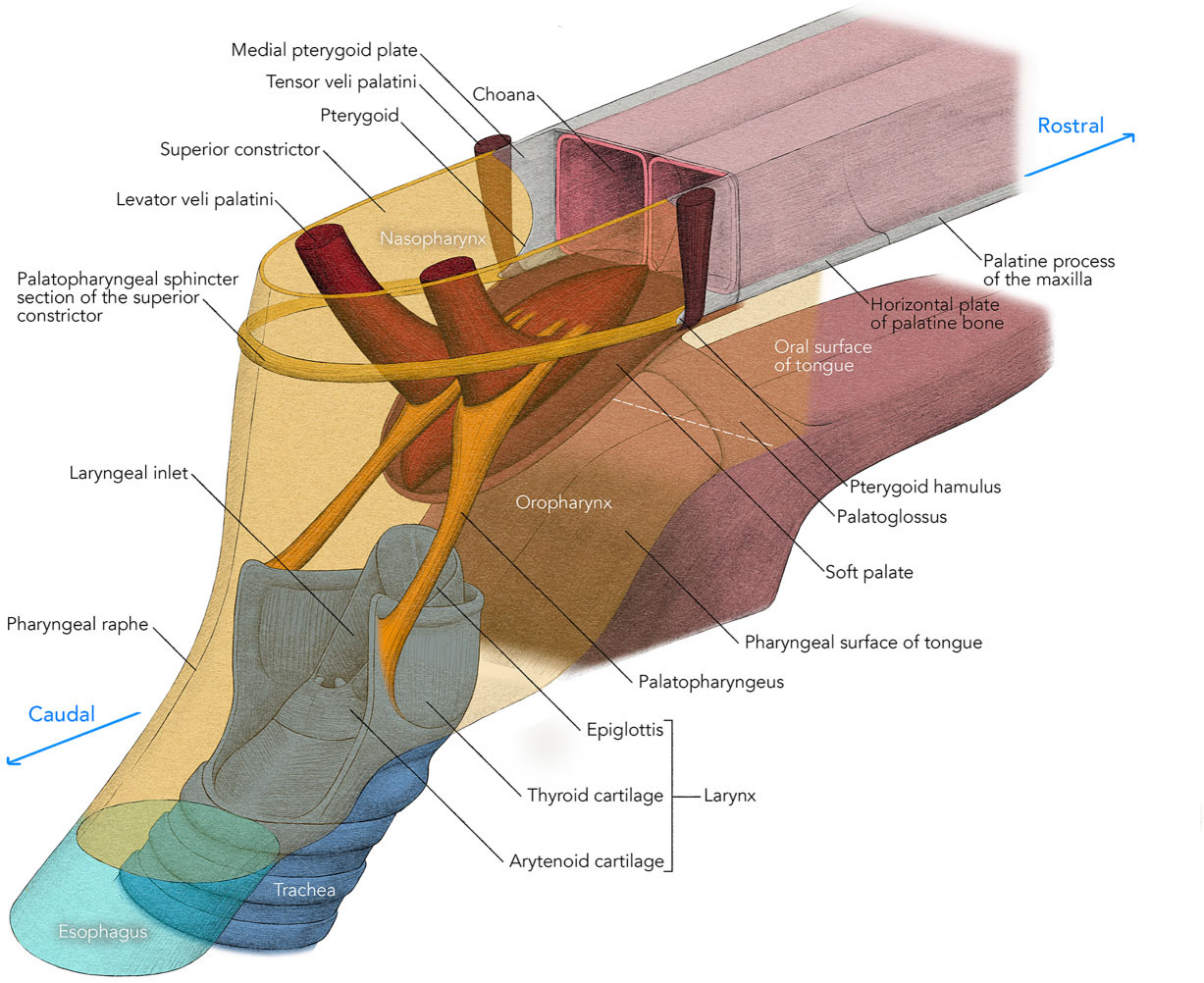
The general arrangement of muscles and structures associated with the soft palate in a typical mammal. Art-work © 2024 Alex Boersma (used with permission).

Four major muscles on each side of the pharynx contribute to the formation and function of the soft palate (Fig. 1). Two (tensor veli palatini and levator veli palatini) originate from the base of the skull and descend into the soft palate from above, while two other muscles, one from the tongue (palatoglossus) and one from the pharynx (palatopharyngeus) ascend into the palate from below. The latter two muscles on each side contribute to the formation of palatoglossal and palatopharyngeal arches that can be observed when the mouth is open. The arms of the palatoglossal arch are lateral and rostral to those of the palatopharyngeal arch and mark the boundary (oropharyngeal isthmus) between the oral cavity and the oropharynx. The oral surface of the tongue forms the floor of the oral cavity, while in most mammals the pharyngeal part of the tongue angles ventrally and is part of the oropharyngeal wall. The palatoglossal and palatopharyngeal muscles depress the soft palate against the elevated back of the tongue and close the oropharyngeal isthmus.

The tensor muscle on each side descends vertically from above and its tendon curves 90° around a bony hamulus on the pterygoid process just inferior and lateral to the posterior openings of the nasal cavity (choana) (Fig. 1). The tendon then expands into an aponeurosis that joins with that from the other side to form a platform into which all the other muscles of the palate insert. The levator veli palatini muscles are not related to hamuli and insert directly into the palate from above and are therefore the only muscles that can elevate the palate beyond the neutral position (Fig. 1).

During swallowing, as described in humans, an anatomically distinct part of the superior constrictor muscle on each side inserts into the soft palate just medial to the hamulus (Whillis 1930) (Fig. 1). This muscle contracts during swallowing and forms a ridge around the pharyngeal wall that catches the soft palate when it elevates. This ridge of muscle, together with the soft palate, closes the pharyngeal isthmus between the nasopharynx and oropharynx, and is termed the “palatopharyngeal sphincter” or “Passavant’s muscle” (Whillis 1930; Prisman and D’Heygere 2021).

In Odontocetes (toothed whales, dolphins, and porpoises), the cranial end of the larynx is telescoped into the nasopharynx and the expanded end is held in position by a muscular sphincter, also termed the “palatopharyngeal sphincter,” first reported as being formed by the palatopharyngeus muscles of the soft palate (Reidenberg and Laitman 1987) and later suggested to be formed by rostral elements of pharyngeal constrictors and the soft palate (Reidenberg and Laitman 2025). As a result, the airway is completely separated from the oral pathway and food passes around one side or the other of the larynx. Because different muscular elements have been reported to be involved in forming the sphincter around the cranial end of the larynx and thereby isolating the nasopharynx from the oropharynx, the status of the soft palate, its relationship to the sphincter, and its function during swallowing and breathing in this group of mammals is not entirely clear.

Based on imaging and dissections of selected small odontocetes, we describe the muscles of the soft palate and present evidence that the sphincter is predominantly formed by part of the superior constrictor muscle on each side that fuses with the soft palate rostrally to anchor the cranial end of the larynx into position within the nasopharynx. This part of the superior constrictor is brought into position near the midline on each side by the presence of a horizontal shelf (palatine surface or plate) at the ventral end of the medial lamina of pterygoid (Mead and Fordyce 2009). This shelf is just caudal to the palatine bone of the hard palate where the soft palate normally attaches in other mammals. We discuss the anatomical findings in the context of sphincter/soft-palate function during swallowing and breathing.

## Materials and methods

The animals included in this study were: (1) an adult male Pacific white-sided dolphin (*Aethalodelphis obliquidens*) of 194 cm total length found live stranded and moribund with gross evidence of physical trauma, rescued from West Vancouver, British Columbia by Vancouver Aquarium staff, and subsequently died, and (2) two harbour porpoises (*Phocoena phocoena*) that were both beach cast and found dead. One of the porpoises was an adult male (138 cm total length) that was recovered from North Saanich, British Columbia on July 16, 2025. The other was a male neonate (94 cm total length) that was collected at Mill Bay, British Columbia, on December 2, 2024. All the specimens were frozen at −20°C and thawed at room temperature prior to analysis. Animals were collected under Fisheries and Oceans Canada licence number XMMS 2 2021.

The pacific white sided dolphin was imaged at the UBC MRI Research Centre using a Phillips 3.0T MR7700 scanner. The DICOM files were uploaded to OsiriX MD (version 14.2.1) and analyzed and final images collected using the 3D Curved-MPR function.

The dolphin, after imaging, and the two harbor porpoises were necropsied by conventional techniques and dissected at the Institute for Oceans and Fisheries at the University of British Columbia, Vancouver, BC.

Two skulls (UBCBBM CTC M017158 and UBCBBM CTC M017921) of adult pacific white-sided dolphins in the collection of the Beaty Museum at the University of British Columbia were evaluated for muscle attachments and photographed as part of the study.

## Results

### Compartments and landmarks

**Fig. 2:**
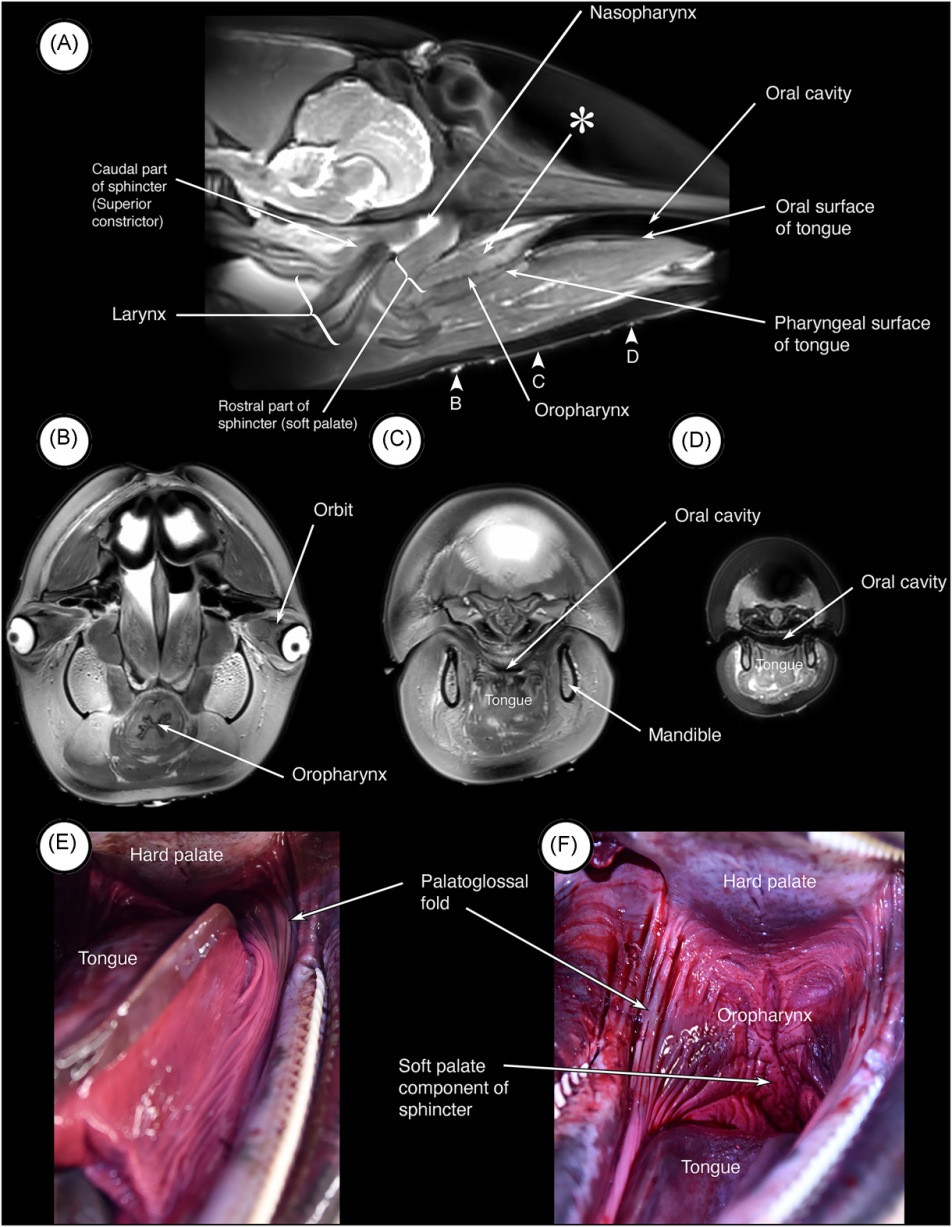
Compartments and landmarks in MRI images and dissection of a pacific white sided dolphin (*A. obliquidens)*. (A) MRI scan in the mid-sagittal plane showing the relevant structures and compartments in the head and neck. The thickened roof of the oropharynx is indicated by the arrow labelled with the asterisk. The arrowheads labelled B, C, and D indicate the positions of the MRI scans in the transverse plane shown in (B), (C) and (D). (E & F) Shown in these images are the palatoglossal folds that mark the lateral margins of the oropharyngeal isthmus.

**Fig. 3:**
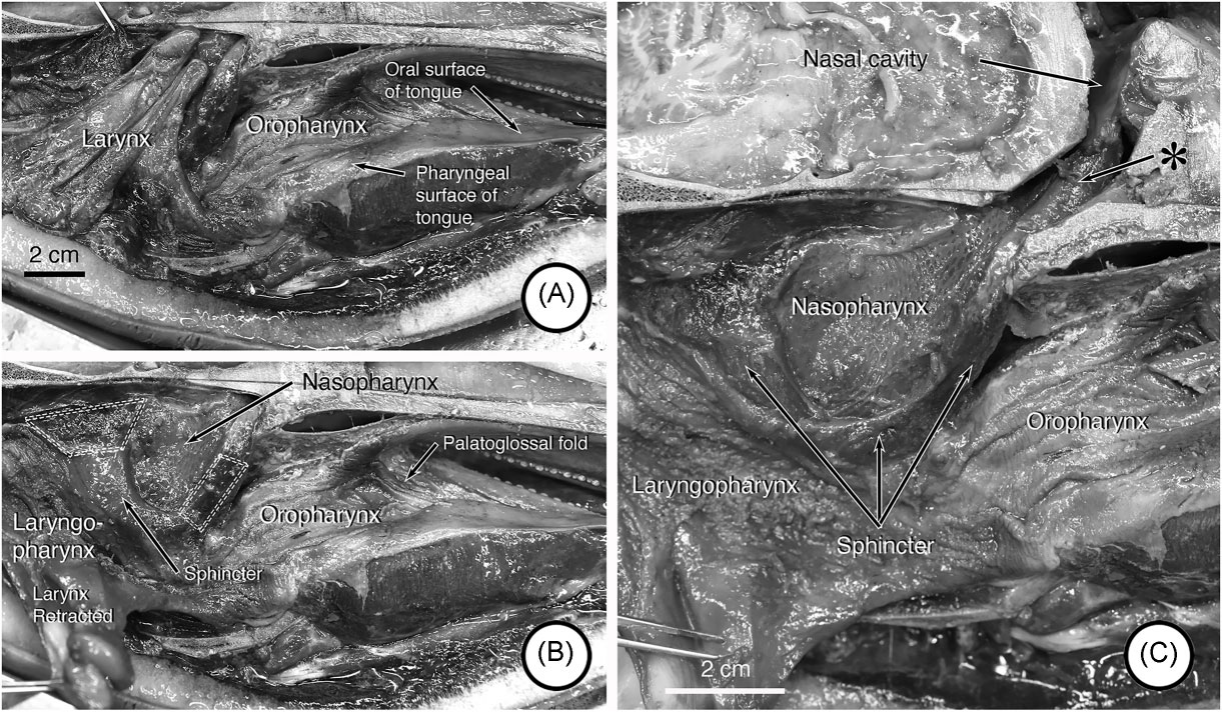
Compartments and landmarks in a dissection of a harbour porpoise (*P. phocoena*). (A) Mid-sagittal view of the head and neck with all structures in position. (B) Mid-sagittal view with the larynx retracted to reveal the nasopharynx and the palatopharyngeal sphincter. The areas outlined by the hatched lines indicate where the sphincter has been cut in the mid-sagittal plane. (C) Mid-sagittal view with the right nasal cavity opened to reveal a muscle (levator veli palatini) (arrow labelled with the asterisk) descending from high on the lateral wall of the cavity and inserting into the rostral end of the palatopharyngeal sphincter.

Components of and general landmarks defining the various compartments and features of the head and neck were visible in the MRI images and dissections. In midsagittal (Fig. 2A) MRI images, the nasopharynx, oral cavity and larynx are apparent. Also visible are the soft tissue structures related to and surrounding the cranial end of the larynx. The oral surface of the tongue is clearly visible in the image and marks the ventral surface of the oral cavity. More caudally, the surface of the tongue appears to slope somewhat ventrally, and tissues associated with the skull in this area thicken (asterisk and arrow in Fig. 2A) and merge caudally with the cranial part of the sphincter anterior to the epiglottis of the larynx. The central cavity in this region is the oropharynx. In transverse images through the oropharynx (Fig. 2B) and oral cavity (Fig. 2C and D), the oropharynx is tubular in appearance and surrounded by a continuous muscular wall whereas the oral cavity has a well- defined tongue ventrally and hard palate dorsally. In dissections of the dolphin (Fig. 2E and F), the palatoglossal folds (arch) mark the oropharyngeal isthmus and the transition from oral cavity to oropharynx. When the head and neck of one of the porpoises is sectioned in the midsagittal plane (Fig. 3) these features also are observed and are consistent with those observed in the dolphin. Particularly striking is the difference between the oral and pharyngeal surfaces of the tongue, and the corrugated appearance of the oropharyngeal mucosa (Fig. 3A). When the larynx (Fig. 3B) is retracted, the laryngopharynx and nasopharynx are evident, as is the sphincter that anchors the cranial end of the larynx in the nasopharynx (Fig. 3C). When the medial wall of one of the nasal cavities is dissected away, a muscle (asterisk and arrow in Fig. 3C) is observed descending from high on the lateral wall into the rostral end of the palatopharyngeal sphincter.

**Fig. 4:**
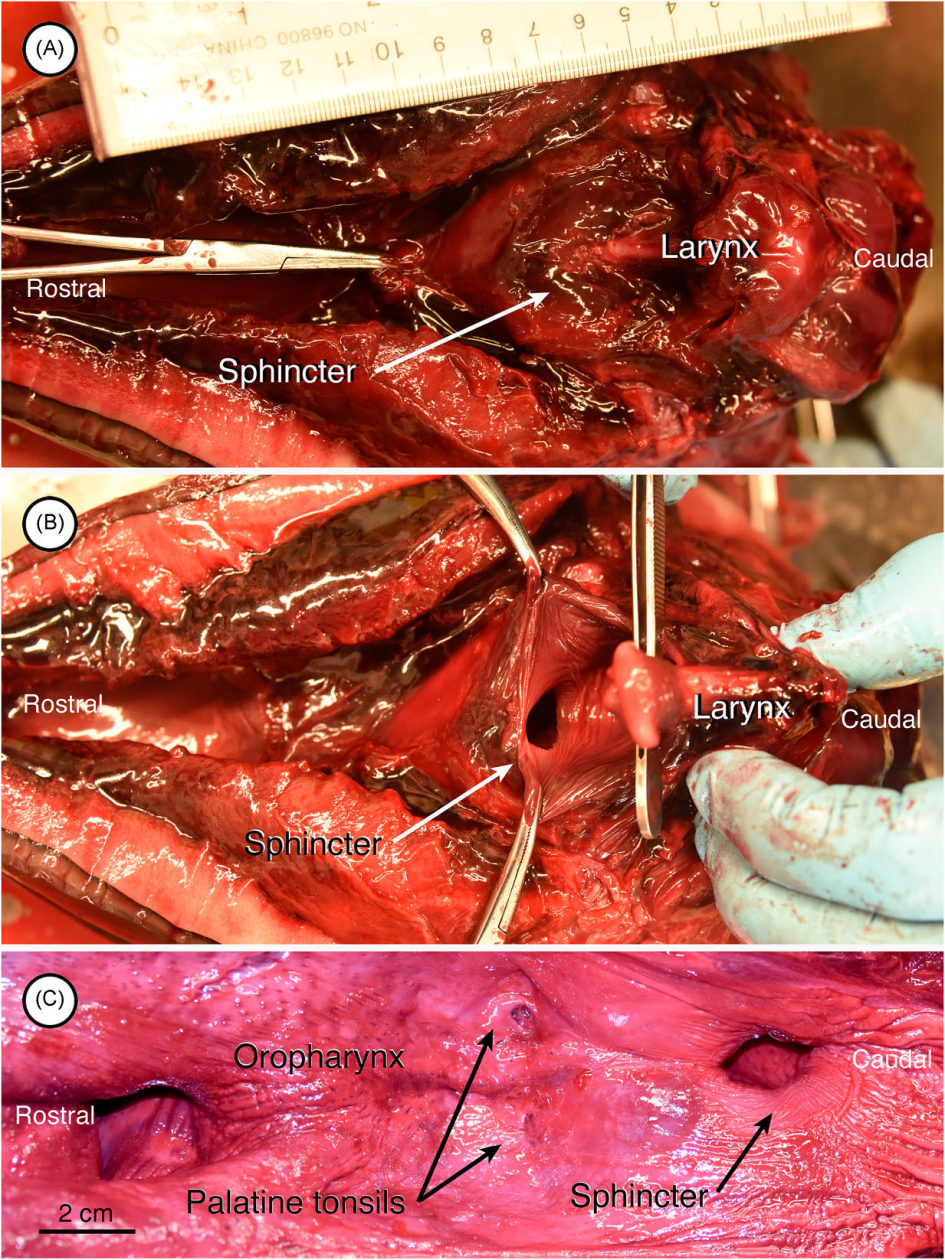
Ventral view of the palatopharyngeal sphincter and related areas in dissections of a harbor porpoise (A, B) and pacific white sided dolphin (C). In these images, the rostral position is on the left and caudal is on the right. (A) In this image, the lower jaw and floor of the oral cavity have been removed, as has mucosa on the roof of the oral cavity and oropharynx. The cranial end of the larynx is enclosed by the sphincter and positioned out of view in the nasopharynx. (B) The cranial end of the larynx has been retracted from the sphincter in this image. (C) Shown here are palatine tonsils rostral to the sphincter in the roof of the oropharynx with the mucosa intact.

When the lower jaw and floor of the oral cavity and oropharynx are removed in one of the porpoises (Fig. 4A), the ventral aspect of the sphincter that holds the cranial end of the larynx into position is evident. This feature is more clearly apparent when the larynx is withdrawn from the sphincter (Fig. 4B). In a similar view of the adult dolphin’s head and neck, palatine tonsils are evident in the roof of the oropharynx (Fig. 4C).

### Muscular components

#### Superior constrictor

**Fig. 5:**
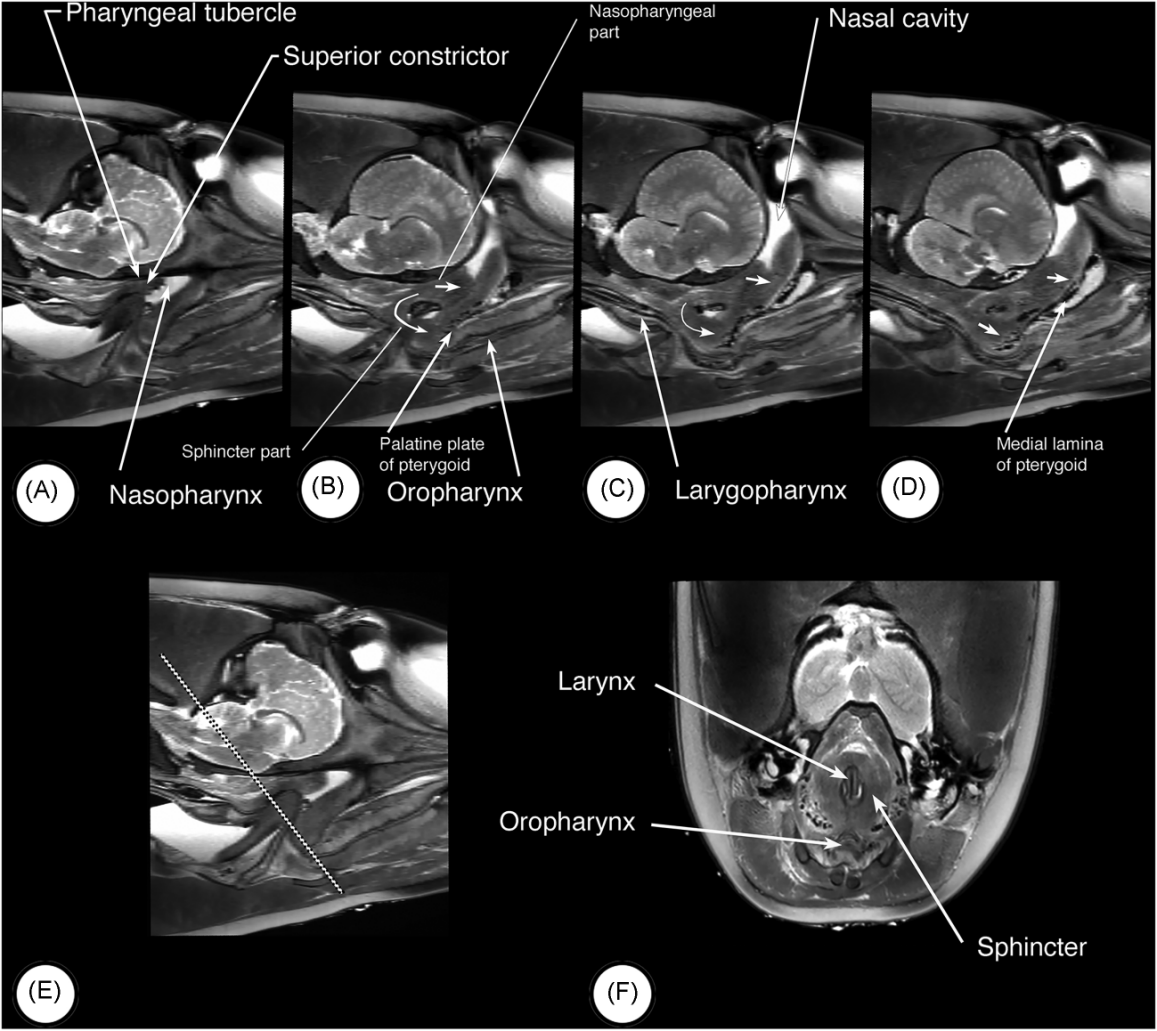
MRI scans showing the position and parts of the superior constrictor of the pharynx in a pacific white sided dolphin. The scans begin in the mid-sagittal plane (A) and progress laterally in (B), (C), and (D). The pharyngeal tubercle in (A) marks the approximate site of origin of the superior constrictor. As the muscle is followed in the sequential scans shown in (B) through (D), it has two parts: a nasopharyngeal part that attaches to the vertical aspect of the medial lamina of the pterygoid and is a component of the nasopharyngeal wall, and a sphincter part that is more ventral, forms a major component of the palatopharyngeal sphincter, merges with the soft palate and attaches to the horizontal part of the pterygoid process. The hatched line in the mid-sagittal section shown in (E) is the approximate position of the oblique section shown in (F). In (F), the sphincter surrounding the rostral end of the larynx just ventral to laryngeal inlet is evident.

**Fig. 6:**
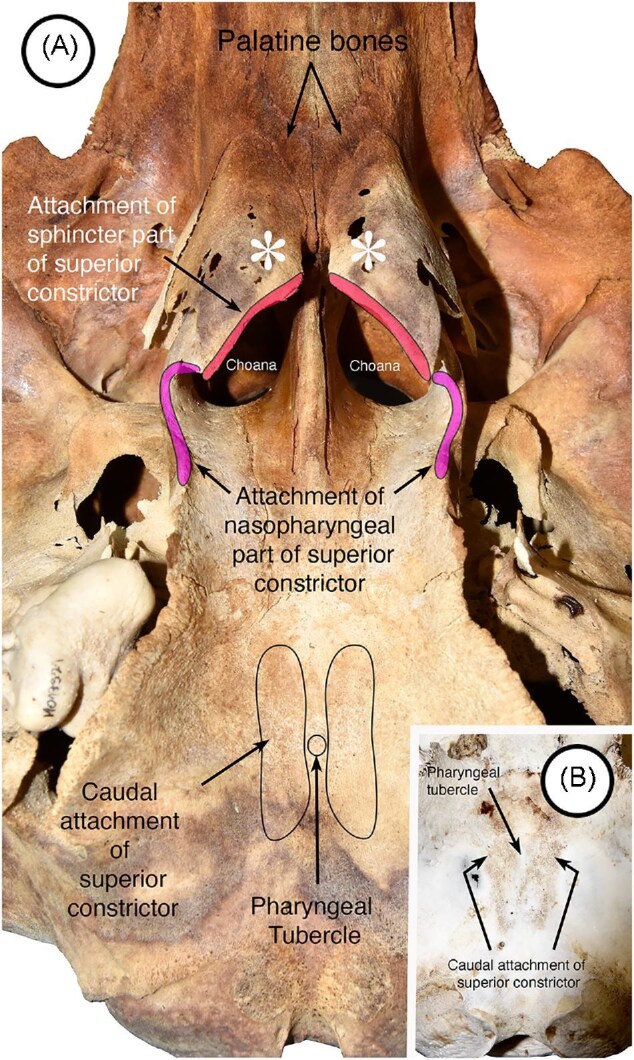
Superior constrictor attachments to bones of the skull from two pacific white sided dolphins. (A) Ventral view showing the caudal (bottom) and more rostral (top) attachments of the muscle. The palatine plates of the pterygoid are indicated by the asterisks. The presence of these plates brings the attachment of the sphincter components of the superior constrictor toward the midline. Note the position of the palatine bones that in most other mammals are usually the end of the hard palate and the attachment sites for the soft palate. (B) Ventral view of the base of the occipital bone showing the approximate position of the pharyngeal tubercle together darkened areas just lateral to the tubercle that correspond to areas in the MRI scans from which the superior constrictor originates.

In midsagittal MRI images, the attachment of the pharyngeal raphe to the pharyngeal tubercle at the midline marks the position of the attachment of the superior constrictor to the base of the occipital bone (Figs. 5A, 6A, B). In sagittal MRI images in progressively more lateral positions, this muscle divides into two components: the more dorsal component (the nasopharyngeal part) expands and passes almost horizontally to attach to the vertical part of the medial lamina of the pterygoid (Fig. 5B–D), whereas the other component (the sphincter part) courses ventrally and rostrally around the cranial end of the larynx to attach to palatine plate of the pterygoid (Fig. 5B–D). The nasopharyngeal part contributes to the lateral wall of the nasopharynx and the sphincter part forms a significant component of the sphincter and rostrally fuses with elements of the soft palate. The attachments of the superior constrictor are shown on skulls of the pacific white-sided dolphin (Fig. 5A and B). When the data set is obliquely sectioned (Fig. 5E), muscle fibers appear to decussate across the midline to surround the rostral end of the larynx (Fig. 5F) just ventral to the expanded lateral flanges of the epiglottis. The palatine plates of the pterygoid to which the sphincter component of the superior constrictor attaches are highlighted by the asterisks in Fig. 6A. The presence of these plates brings the attachment of the sphincter part of the muscle close to the midline on each side.

#### Muscles of the soft palate

##### Tensor veli palatini

**Fig. 7:**
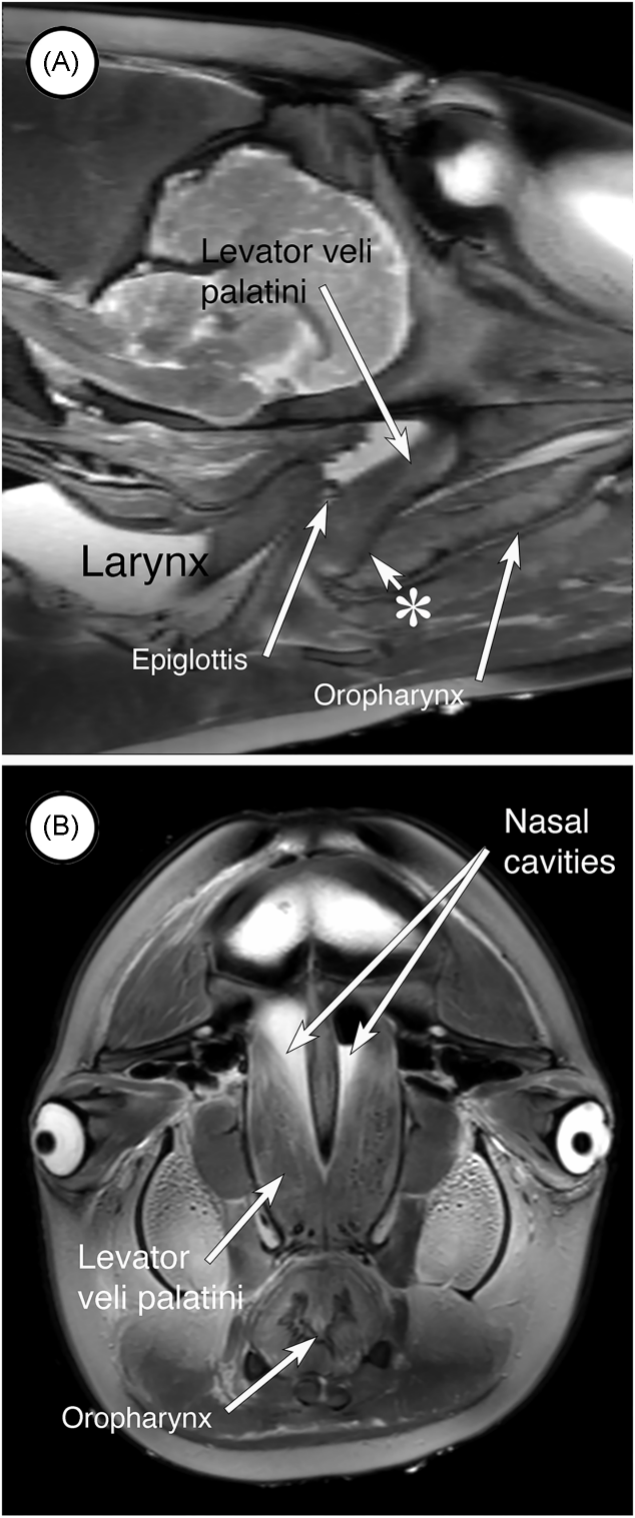
MRI scans in the sagittal (A) and transverse (B) planes showing the levator veli palatini muscles. (A) Shown here is the levator veli palatini muscle emerging from one of the nasal cavities and merging with the rostral aspect of the palatopharyngeal sphincter. A layer of fascia separates (arrow labelled with the asterisk) components of parts of the sphincter rostral to the epiglottis of the larynx and may represent remnants of the aponeurosis of tensor veli palatini. (B) The levator veli palatini muscle on each originates high on the lateral wall of the nasal cavity.

Distinct tensor veli palatini muscles are not apparent in the MRI images nor were they observed in the dissections. It is possible that remnants of the horizontal or aponeurotic components of the muscle are represented by the fascia between the two palatine plates of the pterygoid (not shown) and in the midline septum present in the rostral part of the sphincter (asterisk and arrow in Fig. 7A).

##### Levator veli palatini

Muscles that we tentatively identify as levator veli palatini muscles originate high on the lateral surface of each nasal cavity and descend to merge with the dorsal surface of the sphincter. These muscles are evident in the MRI images of the dolphin (Fig. 7A and B) and were evident in dissections of the porpoise (see Fig. 3C).

##### Palatoglossus

**Fig. 8:**
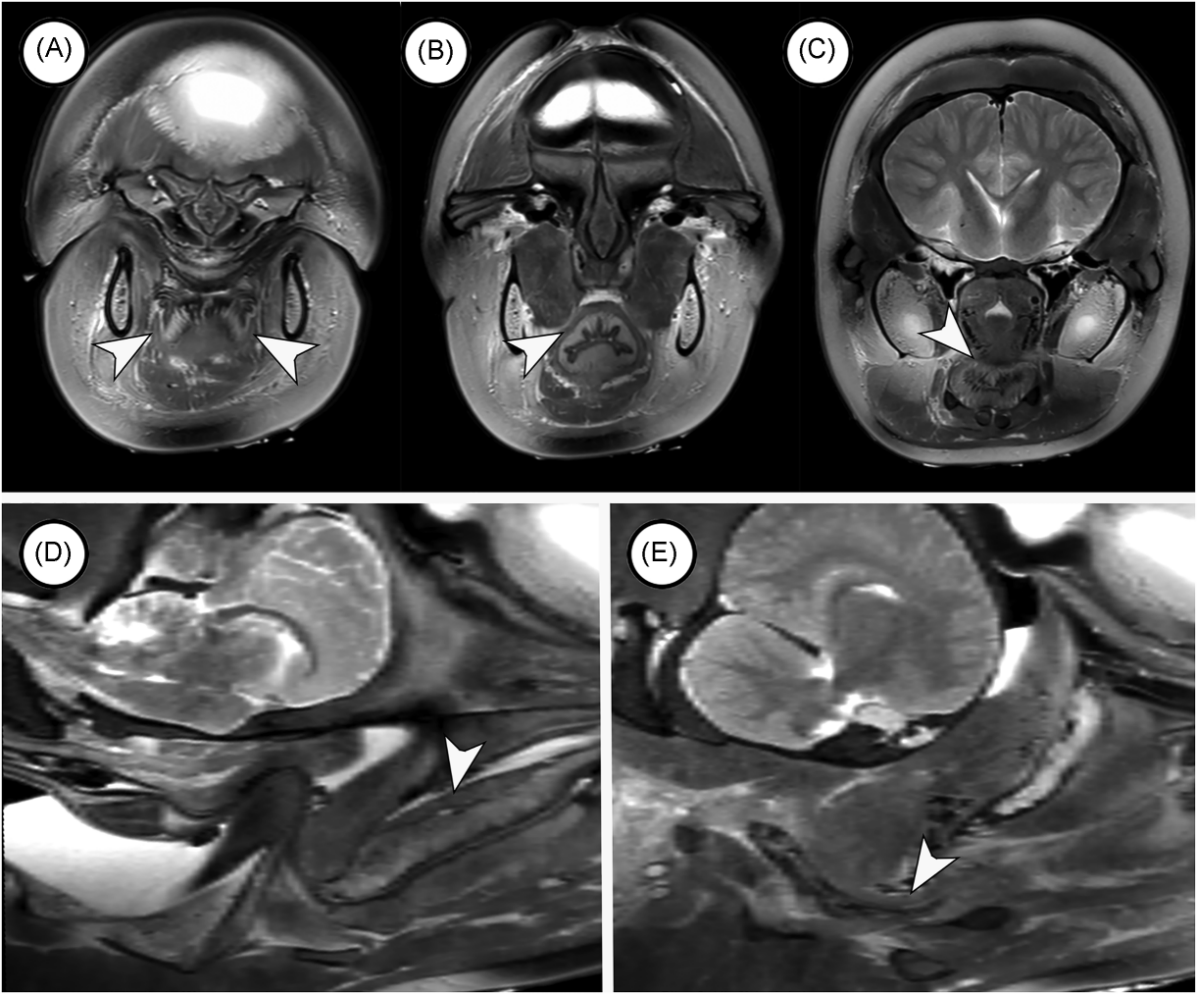
MRI scans in the transverse (A, B, C) and sagittal (C, D) planes showing the distribution of palatoglossus muscles. (A) This scan through the oral cavity shows palatoglossus muscles (arrowheads) on the lateral aspects of the tongue. (B) The palatoglossus muscles become or contribute to a muscular layer that surrounds the oropharynx. (C) This muscle layer merges with the sphincter in caudal regions of the oropharynx. (D) In this midsagittal scan, the muscle layer (arrowhead) associated with the dorsal aspect of the oropharynx merges caudally with the sphincter. (D) In a more lateral sagittal scan, the muscle layer (arrowhead) around the oropharynx merges with the sphincter.

The palatoglossus muscles insert along the lateral surfaces of the tongue (Fig. 8A) and extend posteriorly to merge with or become the prominent muscle layers surrounding the tube-like rostral region of the oropharynx (Fig. 8B). This muscle layer connects with the sphincter caudally (arrowheads in Fig. 8C–E). Because this section of the oropharynx is tubular, contraction of palatoglossus would close the anterior section of the oropharynx when the animal’s mouth is open to catch or manipulate prey.

##### Palatopharyngeus

**Fig. 9:**
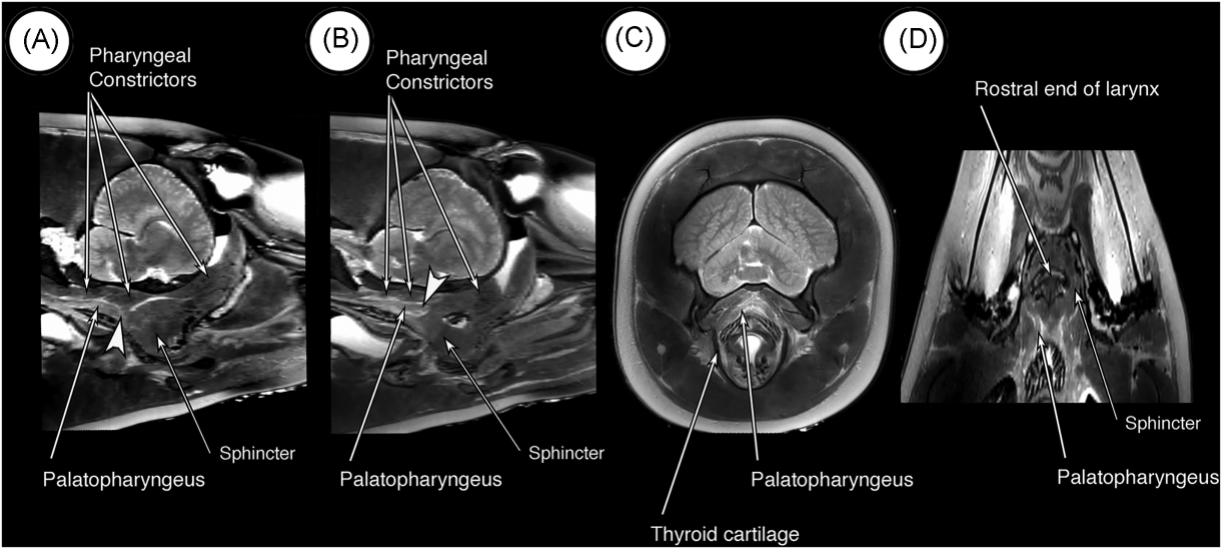
MRI scans in the sagittal (A, B), transverse (C), and dorsal (D) planes showing the palatopharyngeus muscles. (A) The palatopharyngeus muscle on each side is a longitudinal muscle deep to the pharyngeal constrictors. As the muscle inserts into the palatopharyngeal sphincter, it divides into two parts. Shown here is the more lateral part (arrowhead) that inserts ventrally into the sphincter. (B) Shown here is the more medial part of palatopharyngeus as it inserts dorsally into the sphincter. (C) In this transverse scan the muscle attaches to the thyroid cartilage. (D) In this scan in the dorsal plane, the palatopharyngeus muscles are shown inserting into the sphincter.

The palatopharyngeus muscles are present in the MRI images of the dolphin as horizontal bands deep to the constrictor muscles of the pharynx (Fig. 9). Each palatopharyngeus muscle attaches to the caudal aspect of the sphincter on each side via two bands: the lateral band attaches ventrally into the sphincter (arrowhead in Fig. 9A), and the medial band attaches more dorsally (arrowhead in Fig. 9B). Caudally, fibers decussate across the midline with those on the other side and the muscles attach to the posterior laminae of the thyroid cartilage of the larynx (Fig. 9C). In MRI images in the dorsal plane, the palatopharyngeus muscles also are visible deep in the pharyngeal constrictors (Fig. 9D).

## Discussion

Using MRI scans together with dissections of a pacific white-sided dolphin and two harbour porpoises, we show that the “palatopharyngeal sphincter” is formed by fusion of the superior constrictor of the pharynx to the soft palate around the cranial end of the larynx. This sphincter effectively anchors the laryngeal inlet in the nasopharynx and permanently isolates the airway from the gut tube. Muscles of the soft palate position the sphincter during breathing and swallowing. We speculate that the sphincter is most contracted during swallowing to hold the rostral end of the larynx in the nasopharynx as a prey item is moved through the piriform recesses on one side or the other of the larynx and into the esophagus.

### Anatomical findings

**Fig. 10:**
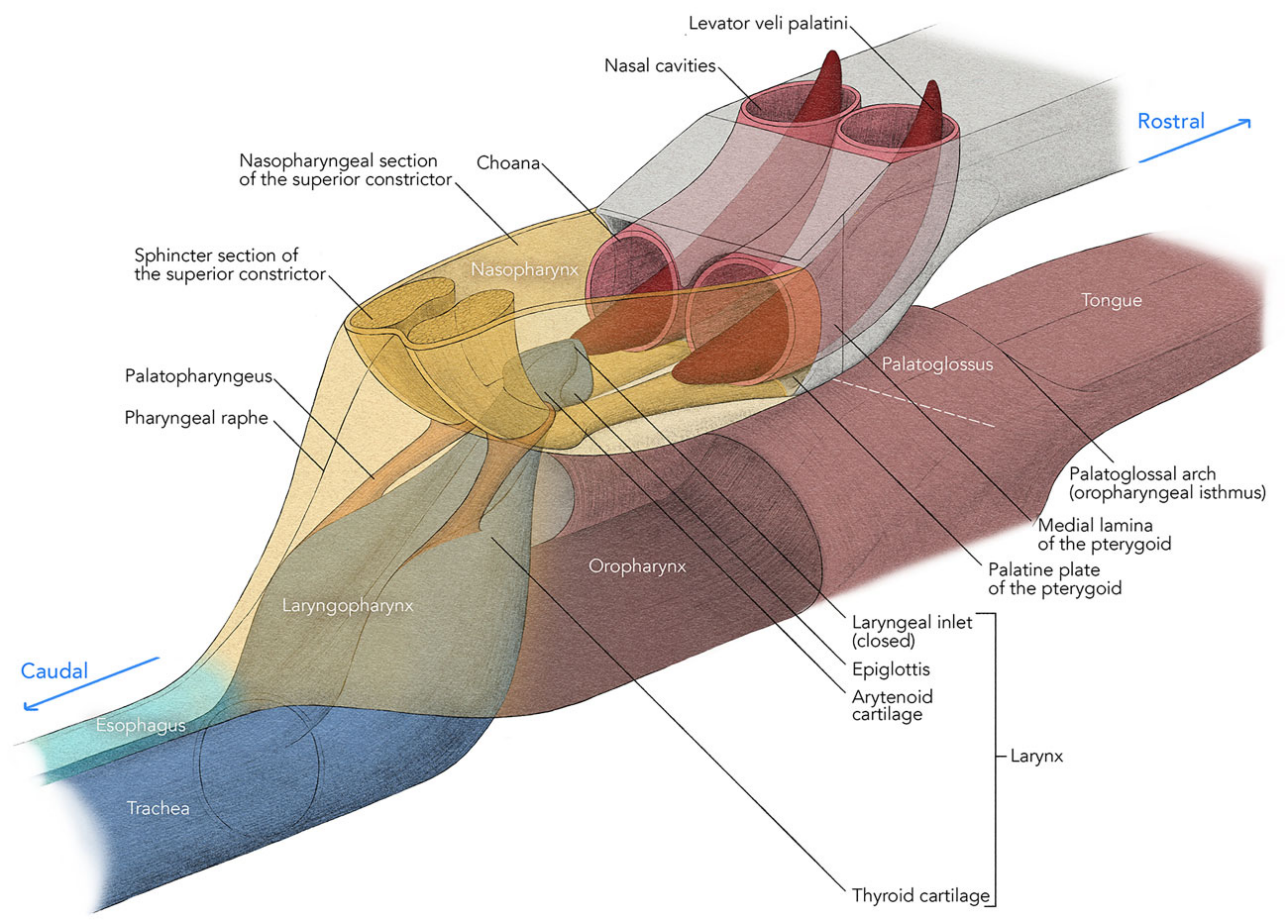
Schematic diagram illustrating the various components of the palatopharyngeal sphincter and related structures. Art-work © 2024 Alex Boersma (used with permission).

The organization of the palatopharyngeal sphincter is summarized in Fig. 10. A significant component of the sphincter on each side is a part of the superior constrictor muscle of the pharynx. This muscle is identified as superior constrictor based on its origin in association with the pharyngeal tubercle on the occipital bone and its rostral attachments to the pterygoid process. In other mammals, the pharyngeal fascia and part of the superior constrictor muscle attaches on each side to the pterygoid process that is just posterior and lateral to the associated choana. In odontocetes, the presence of palatine plates at the ventral ends of the pterygoid brings the rostral attachment of the superior constrictor close to midline so that the muscle body on each side captures the upper end of the larynx that is telescoped into the nasopharynx above the level of the soft palate. This part of superior constrictor on each side merges with the soft palate and together they form the rostral component of the sphincter. It is tempting to speculate that the part of superior constrictor that forms the sphincter in odontocetes is homologous with that part of the superior constrictor muscle in humans that forms a ridge around the pharyngeal wall that catches the soft palate during swallowing and that is also called the palatopharyngeal sphincter (Whillis 1930).

Interestingly, the palatine plates of the pterygoid in odontocetes are positioned caudal to the palatine bones where the hard palate normally ends and where the soft palate is attached in other mammals (Sisson et al. 1975; El Allali et al. 2017; Missagia and Perini 2018; Mohamed 2019; Keneisenuo et al. 2022; Núñez-Cook et al. 2022). This may account for the thickened tissue that appears to include part of the palatoglossus muscle dorsal to the oropharynx and that is attached to the hard palate in this study. In other words, the palatine plates of the pterygoid project into the rostral part of the soft palate, possibly within the original plane of the palatine aponeurosis, and split it into components above and below the plates. The part below the shelf and that is attached to bone becomes part of the roof of the oropharynx and may explain the observation that muscle in this region appears to be part of the palatoglossus muscle.

As in other mammals, the palatoglossus muscle on each side underlies the palatoglossal fold and marks the oropharyngeal isthmus or the transition from oral cavity to oropharynx. This transition also is characterized ventrally by a change in the mucosa of the tongue from oral to pharyngeal. The oropharynx in odontocetes, at least as visualized in MRI images of the dolphin, is distinctly tubular. A significant feature of the oropharynx is the presence of palatine tonsils which we identified just anterior to the sphincter.

In addition to the palatoglossus muscles, which appear to form a significant part of the oropharyngeal wall, other major muscles of the soft palate also are present in odontocetes. These include the palatopharyngeus and levator muscles, and possible remnants of tensor veli palatini muscles. Each palatopharyngeus muscle is oriented horizontally deep to the pharyngeal constrictor muscles and is attached caudally to the thyroid laminae and cranially to the sphincter. As the muscle approaches the sphincter, it divides into two bands; one that attaches dorsally to the sphincter and one that attaches more ventrally.

A prominent muscle that we tentatively identify as levator veli palatini descends from high in each nasal cavity into the sphincter and merges with the superior constrictor. This muscle is particularly evident in the MRI scans of the dolphin and was verified in dissections of the porpoises. The muscle is similar in position to the levator veli palatini identified in fin whales (Vogl et al. 2024), but is positioned much higher in nasal cavities of the dolphin and porpoise. Because in other mammals the levator veli palatini originates more caudally from elements of the temporal bone associated with the ear, the actual homology of the muscle identified as the levator in the Cetacea with that in other mammals needs to be confirmed. Another notable feature of this muscle in odontocetes is that it would tend to block the nasal cavity when contracted, as we predict would happen during swallowing. The muscle would be less occlusive when fully relaxed and lengthened, as we predict would happen during breathing.

Remnants of the palatine aponeurosis, formed by horizontal parts of the tensor veli palatini muscles in other mammals, is likely represented by the fascia in the midline between the unfused parts of the palatine plates of the pterygoid processes and by the extension of this fascia into the rostral part of the sphincter.

### Biomechanical model for breathing and swallowing

**Fig. 11:**
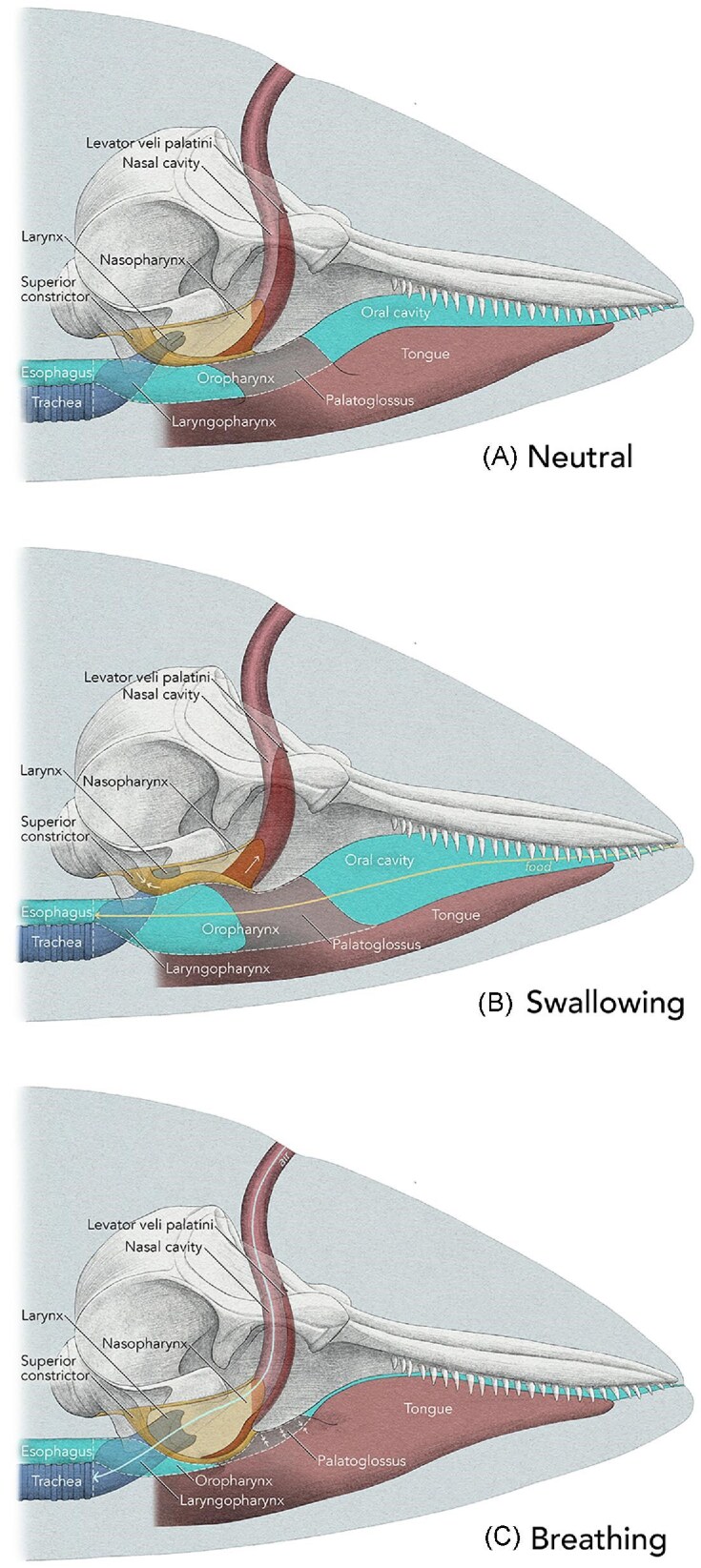
Schematic diagram illustrating components of the palatopharyngeal sphincter (A) and their functions in odontocetes during swallowing (B) and breathing (C). The arrows in (B) and (C) indicate contraction. In (B) the oropharyngeal isthmus is open and the tongue is shown as depressed as a large prey item passes from the oral cavity into the pharynx. It is not known if tongue base retraction and contraction of palatoglossus and palatopharyngeus muscles occur to move prey through the pharynx and into the esophagus in odontocetes. Art-work © 2024 Alex Boersma (used with permission).

The position of the major components of the sphincter in the neutral or rest position, and their hypothesized function during swallowing and breathing are summarized in Fig. 11. Although palatopharyngeus is not shown in this image it extends somewhat horizontally between the thyroid cartilage on each side to the palatopharyngeal sphincter.

In the rest or neutral position, when the animal is not breathing or swallowing, the laryngeal inlet is in the nasopharynx and seemingly held in position by muscle tone in the palatopharyngeal sphincter (Fig. 11A). The palatoglossus muscles surround the oropharynx maintain a small luminal space.

During swallowing (Fig. 11B), initial relaxation of the palatoglossus and palatopharyngeus muscles would allow opening of the oropharyngeal isthmus and passage of food into the oropharynx while the levator and constrictor components of the superior constrictor are maximumly contracted to maintain the rostral end of the larynx in the nasopharynx. This latter is consistent with the motor activity in humans where the pharyngeal isthmus is closed by elevation of the soft palate by the levator muscles and constriction of the palatopharyngeal sphincter component of the pharyngeal wall. As prey passes into the pharynx, it is unknown if contraction of palatoglossus and palatopharyngeus muscles and tongue base retraction facilitate propelling food through the pharynx and into the esophagus as occurs in other mammals (Doty and Bosma 1956; Li et al. 2025). The importance of the palatopharyngeal sphincter in maintaining the laryngeal inlet in the nasopharynx in odontocetes is highlighted by cases in which too large a prey item is swallowed with catastrophic consequences (Watson and Glee 2005; Stolen et al. 2013). In these cases, the laryngeal inlet is dislodged from the sphincter and the animals asphyxiate.

**Fig. 12:**
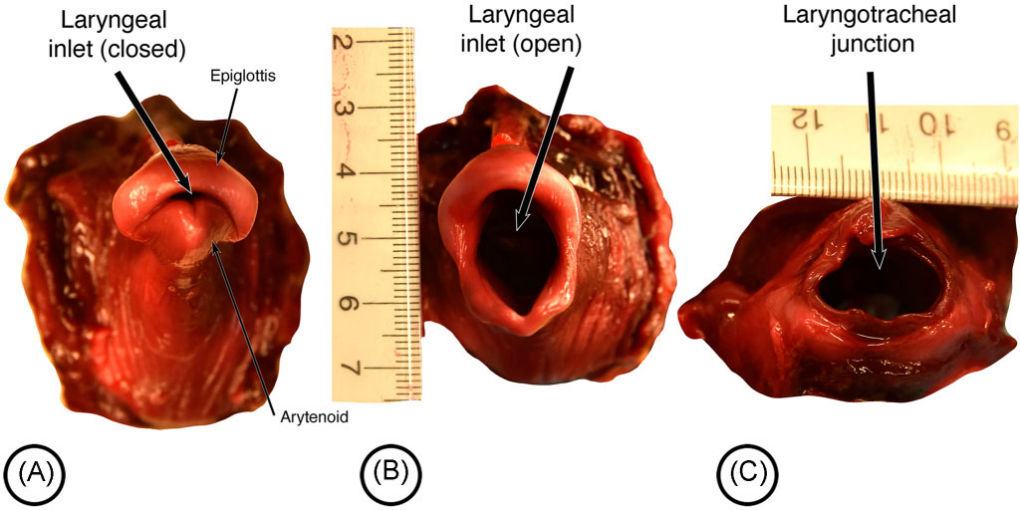
Larynx of a harbour porpoise. (A) Superior view with laryngeal inlet closed. (B) Superior view with laryngeal inlet open. (C) Inferior view of laryngo-tracheal junction. Notice that the open laryngeal inlet is approximately the same dimensions as the lumen of the laryngotracheal junction.

During breathing, we speculate that the sphincter and the levator relax to allow the laryngeal inlet to fully open (Fig. 11C). When not breathing, which is most of the time, the laryngeal inlet is closed or in the rest position (as shown in Fig. 12A). When breathing, the laryngeal inlet is maximally opened as illustrated on an isolated porpoise where the inlet has been opened artificially (Fig. 12B). Significantly, the opened laryngeal inlet approximates the same diameter as the laryngo-tracheal junction (Fig. 12C), which suggests unrestricted airflow through the larynx when breathing. Although homologues of vocal and vestibular folds have been identified within the laryngeal cavity itself (Reidenberg and Laitman 1988), the folds appear vestigial, are oriented parallel to airflow, and likely also do not restrict airflow when breathing. In addition, relaxation and elongation of the levator muscles would reduce their diameters and facilitate airflow through the nasal cavities. The palatoglossus muscles and palatopharyngeus muscles may contract to pull the rostral part of the sphincter forward and the caudal part backwards respectively to facilitate dilating the sphincter. This would correspond to the motor pattern seen in terrestrial mammals when the palate is depressed by palatoglossus and palatopharyngeus to close the oropharyngeal inlet so that food can be manipulated in the oral cavity while breathing.

## Summary

Dynamic separation of the food and air pathways in terrestrial vertebrates is and has been a necessary and fundamental characteristic of the aerodigestive tract ever since the evolution of the lower airway from the gut tube. In mammals, the soft palate together with components of the larynx function as valves to ensure that food and liquid do not enter the airway during swallowing. Odontocete cetaceans are unique in that the opening to the lower airway (the laryngeal inlet) is anchored into the nasopharynx by a “palatopharyngeal sphincter,” thereby permanently separating the airway from the gut tube (Reidenberg and Laitman 1987). Here we show that the palatopharyngeal sphincter of the pacific white sided dolphin and the harbour porpoise is formed by the fusion of part of the superior constrictor muscle of the pharyngeal wall on each side to the soft palate. The development of palatine plates from the ventral end of the pterygoid bone on each side moves the rostral attachment of the superior constrictor close to the midline, caudal to the end of the horizontal plates of the palatine bones where the hard palate normally ends in other mammals. As the muscle fibers course from caudal to rostral, they appear to decussate around the rostral end of the larynx anchoring the laryngeal inlet in the nasopharynx, and then the muscles merge with elements of the soft palate. The four major muscles, or their remnants, of the soft palate are present and help form or insert into the sphincter. The palatoglossus muscles are prominent, underlie the palatoglossal folds and form major components of the oropharyngeal wall prior to attaching into rostral parts of the sphincter. Also present are palatopharyngeus muscles that course almost horizontally from the thyroid laminae of the larynx to the sphincter. Possible remnants of the palatine aponeurosis formed by the tensor veli palatini muscles are represented by the fascial layer between the unfused palatine plates of the pterygoid bone and the layer of fascia that separates the rostral soft palate component of the sphincter into dorsal and ventral parts. Muscles we tentatively identify as levator veli palatini muscles attach high on the lateral aspect of each nasal cavity and descend to insert into the sphincter. We predict that the sphincter (superior constrictor part and levator veli palatini) is most constricted during swallowing to firmly anchor the laryngeal inlet in the nasopharynx while “large” prey items pass through the piriform recess on one side or the other of the larynx. During breathing, the sphincter is more relaxed to enable the laryngeal inlet to maximally open and allow unrestricted airflow, and the palatoglossal and palatopharyngeal muscles contract to facilitate opening the sphincter along the cranio-caudal axis.

## Data Availability

The data underlying this article will be shared on reasonable request to the corresponding author.
